# Prediction of hearing recovery in unilateral sudden sensorineural hearing loss using artificial intelligence

**DOI:** 10.1038/s41598-022-07881-2

**Published:** 2022-03-10

**Authors:** Min Kyu Lee, Eun-Tae Jeon, Namyoung Baek, Jeong Hwan Kim, Yoon Chan Rah, June Choi

**Affiliations:** 1 Department of Otorhinolaryngology-Head and Neck Surgery, Korea University Ansan Hospital, College of Medicine, Korea University, 123, Jeokgeum-ro (Gojan-dong), Danwon-gu, Ansan-si, Gyeonggi-do 15355 Republic of Korea; 2 Department of Neurology, Korea University Ansan Hospital, College of Medicine, Korea University, Ansan, Republic of Korea; 3 Medical Science Research Center, Korea University Ansan Hospital, Korea University College of Medicine, Ansan, Republic of Korea

**Keywords:** Diseases, Medical research, Risk factors

## Abstract

Despite the significance of predicting the prognosis of idiopathic sudden sensorineural hearing loss (ISSNHL), no predictive models have been established. This study used artificial intelligence to develop prognosis models to predict recovery from ISSNHL. We retrospectively reviewed the medical data of 453 patients with ISSNHL (men, 220; women, 233; mean age, 50.3 years) who underwent treatment at a tertiary hospital between January 2021 and December 2019 and were followed up after 1 month. According to Siegel’s criteria, 203 patients recovered in 1 month. Demographic characteristics, clinical and laboratory data, and pure-tone audiometry were analyzed. Logistic regression (baseline), a support vector machine, extreme gradient boosting, a light gradient boosting machine, and multilayer perceptron were used. The outcomes were the area under the receiver operating characteristic curve (AUROC) primarily, area under the precision-recall curve, Brier score, balanced accuracy, and F1 score. The light gradient boosting machine model had the best AUROC and balanced accuracy. Together with multilayer perceptron, it was also significantly superior to logistic regression in terms of AUROC. Using the SHapley Additive exPlanation method, we found that the initial audiogram shape is the most important prognostic factor. Machine/deep learning methods were successfully established to predict the prognosis of ISSNHL.

## Introduction

Idiopathic sudden sensorineural hearing loss (ISSNHL) refers to an abrupt onset of hearing loss at > 30 dB for at least three contiguous frequencies within 72 h^1^. This is a common otologic emergency with an incidence of 5–20 cases per 100,000 persons annually^2^. Most cases are idiopathic and the pathogenesis of the disease remains debatable. Principal theories for ISSNHL include viral infection, vascular occlusion, intracochlear membrane breaks, and autoimmunity^1,3^. Since the fundamental mechanisms of ISSNHL are poorly understood, its treatment is controversial. However, steroid therapy, including systemic, intratympanic, or both, has become the most widely accepted treatment option^1,4^. In addition, due to the unpredictable course of ISSNHL, several variables that appear to influence the prognosis of ISSNHL have been identified. These include the severity of hearing loss, audiogram shape, presence of vertigo, and age^2,3,5–9^.

Creating optimization models to predict a prognosis by analyzing various factors using artificial intelligence as well as selecting important variables can be an innovative method in any medical field. Artificial intelligence has been widely applied in the field of audiology. For example, it has been used to predict hearing loss in industrial workers exposed to noise, as well as the prognosis of ISSNHL^10–12^. In a previous report, predictive models based on four machine learning methods including deep belief network (DBN), logistic regression (LR), support vector machine (SVM), and multilayer perceptron (MLP) have been applied in ISSNHL with the outcomes of 1220 patients^13^. The DBN model provided the best predictive ability, achieving an accuracy of 77.58% and an area under the receiver operator characteristic curve (AUROC) of 0.84^13^.

In another study, several prediction models with machine learning methods, including LR, least absolute shrinkage and selection operator, decision tree, random forest (RF), SVM, and boosting were developed^14^. With the medical data of 244 patients, the RF method achieved the highest predictive power with an accuracy of 72.22% and an AUROC of 0.7445. The importance of variables using the Gini index was also evaluated^14^. In addition, in our previous study, machine learning methods including adaptive boosting, K-nearest neighbor, MLP, RF, and SVM were used with the data from 227 patients^15^. The SVM model using selected predictors showed the best performance with an accuracy of 75.36% and an AUROC of 0.76^15^. However, in the current study, we evaluated important variables using SHapley Additive exPlanation (SHAP). This study aimed to assess new important variables and increase the performance of machine learning/deep learning models for predicting hearing recovery in patients with ISSNHL after 1 month of treatment.

## Materials and methods

### Study population and data collection

The medical records of 813 patients with unilateral ISSNHL who underwent treatment at our hospital between January 2010 and December 2019 were retrospectively reviewed. The diagnostic criterion for ISSNHL was sudden hearing loss (30 dB or more) for at least three contiguous frequencies within 72 h. ISSNHL with clear etiologies, including vestibular schwannoma, were excluded. Finally, 453 patients who had pure tone audiometry (PTA) data at the beginning and 1 month after treatment were included.

Patients were treated with either systemic steroids (e.g., methylprednisolone 64 mg, tapering for 14 days or dexamethasone 5 mg intravenously three times/day for 4 days and then tapered over 8 days), intratympanic dexamethasone injection (1–4 times), or both. Patients underwent PTA on their first visit and one month after treatment. Hearing thresholds at 0.125 kHz, 0.25 kHz, 0.5 kHz, 1 kHz, 2 kHz, 3 kHz, 4 kHz, and 8 kHz were measured for PTA. Recovery after one month was defined according to Siegel’s criteria as follows: (1) complete recovery included final hearing levels better than 25 dB; (2) partial recovery was > 15 dB gain and final hearing levels between 25 and 45 dB; (3) slight recovery was > 15 dB gain and final hearing was poorer than 45 dB; and (4) no improvement was < 15 dB gain, or final hearing was poorer than 75 dB^16^. In the present study, the hearing threshold was determined as the average of four frequencies (500 Hz, 1000 Hz, 2000 Hz, 4000 Hz). Patients with “complete recovery” and “partial recovery” according to Sigel’s criteria were considered to be in the recovery group. However, patients with “slight recovery” and “no improvement” were considered to be in the no-recovery group. The variables were extracted from demographic data, medical records, pure-tone audiometry, and laboratory data. According to the shape, the pure-tone audiometry was classified into five types: ascending, U-shaped, descending, flat, and deaf. A list of all collected data and the number of missing values for each variable are listed in Supplementary Tables S1 and S2.

### Data splitting and preprocessing for outlier detection/imputation

Binary variables with less than 80% of missing values and multinomial and numeric variables with less than 60% of missing values were included^17^. First, 25% of the data was randomly separated by stratification of ISSNHL. It was used as a test dataset for the evaluation of the final model only. The remaining 75% of the data was used for the model construction processes using the leave-one-out cross-validation strategy. Outliers were detected using isolation forest^18^. They were replaced with the closest non-outlier value within the training set. Imputation for continuous variables was performed using multivariate imputation chained equations (MICE)^19^. The imputation was limited to the bounds of the training set. The imputed values for the discrete-value variables were rounded to the nearest integer.

### Feature selection and feature importance analyses

First, features were evaluated for their contribution to the model prediction and selected using recursive feature elimination. This achieved the highest performance of the AUROC during cross-validation^20^. After the recursive feature elimination, the selected features were used to build the prediction models. The evaluated features are shown in Supplementary Table S1. The contribution of each variable to the model’s performance was evaluated using the mean absolute SHAP value with LightGBM, which is a gradient boosted tree model^21^. LightGBM can handle categorical variables unlike other algorithms. This can be advantageous in avoiding overfitting. The SHAP value provides directionality of the contribution of each variable’s value to the model's decision using positive and negative values. An additional stepwise process was performed during the recursive feature elimination to minimize the effect of multicollinearity. This can cause underestimation of the relative importance of variables^22^. Hierarchical double clustering was applied during recursive feature elimination. In every recursion, features were clustered twice on their Spearman rank-order correlations. Each feature was evaluated with a new feature set, including features within other clusters. There were no clusters with a Ward’s linkage of less than one after double clustering.

### Modeling

We selected one conventional statistical model, logistic regression, as a baseline comparator. We also selected four popular machine learning and deep learning models: support vector machine^23^, light gradient boosting machine (LightGBM)^24^, extreme gradient boosting (XGBoost)^25^, and multilayer perceptron (MLP)^26^.

Bayesian optimization, which makes a surrogate model of an acquisition function, was used to determine the best promising hyperparameters that maximized the AUROC in the cross-validation scheme.

**Figure 1:**
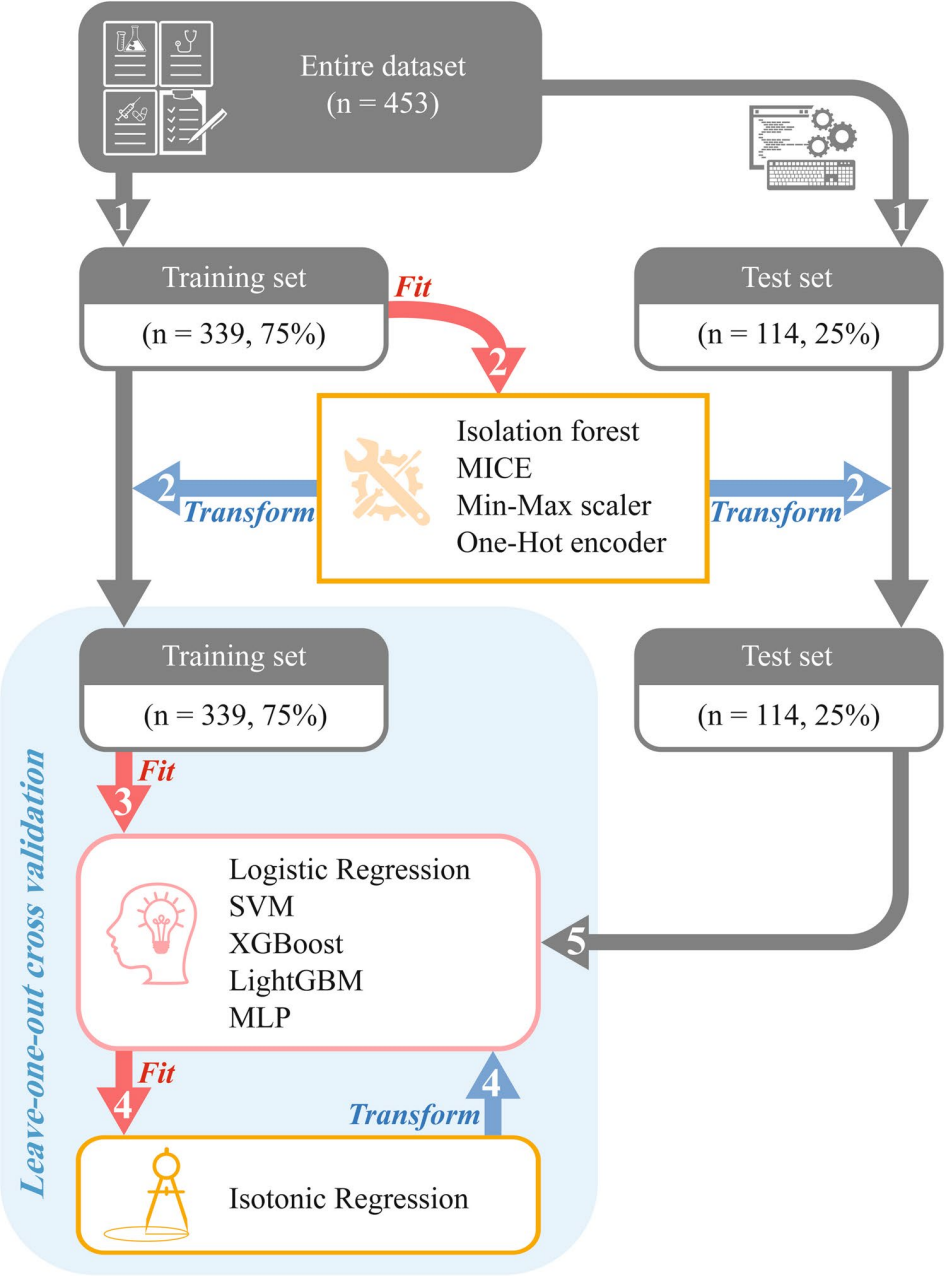
Flowchart for developing predictive models. Numbers above the arrows indicate the order of the processes: (1) data stratified random splitting, (2) data preprocessing, (3) model construction, (4) model calibration, and (5) performance evaluation. *MICE* multivariate imputation by chained equation, *SVM* support vector machine, *XGBoost* extreme gradient boosting, *LightGBM* light gradient boosting machine, *MLP* multilayer perceptron.

In MLP training, an early stopping strategy, batch normalization^27^, and dropout^28,29^ were used to prevent overfitting. Glorot uniform initializer^30^ was used to initialize the activation function; and the Nesterov Adam optimizer^31^ was used to optimize the weight parameters. All processes were implemented in Python 3.8.2, using TensorFlow-GPU 2.4.0. A flowchart for developing predictive models is shown in Fig. 1.

### Primary outcome and evaluation criteria

The primary outcome metric for model performance was chosen as the AUROC. Cross-validation and early stopping were performed to maximize the AUROC. The constructed models were evaluated on how often they were confident and how often the were wrong, using a threshold of 0.50.

### Statistical analysis

Descriptive statistics were presented as the number (%), mean (SD), or median (25% and 75% interquartile values). The Shapiro–Wilk test for normality and Levene’s test for homoscedasticity were used. Comparison analysis was performed using the chi-square test, independent *t-*test, or Mann–Whitney U test.

The AUROC was calculated and compared between models using Delong's method^30^. In the multiple comparison of AUROC, probability values were adjusted using Bonferroni correction. The AUPRC with a 95% CI was also calculated. The calibration error was evaluated using the Brier score, which is the mean squared error for the predicted probability^32^. The balanced accuracy and F1 score, which is a weighted average of the precision and recall, was calculated. The significance level was set at P < 0.05.

### Ethical approval

This study was approved by the Institutional Review Board of the Korea University College of Medicine (IRB. No. 2020AS0174) and informed consent is waived by ethics committee along with the Institutional Review Board of the Korea University College of Medicine. The study followed the Transparent Reporting of a multivariable prediction model for Individual Prognosis or Diagnosis (TRIPOD) reporting guidelines^33^. All methods were performed in accordance with relevant guidelines and regulations.

## Results

### Clinical characteristics and features according to recovery status

**Figure 2:**
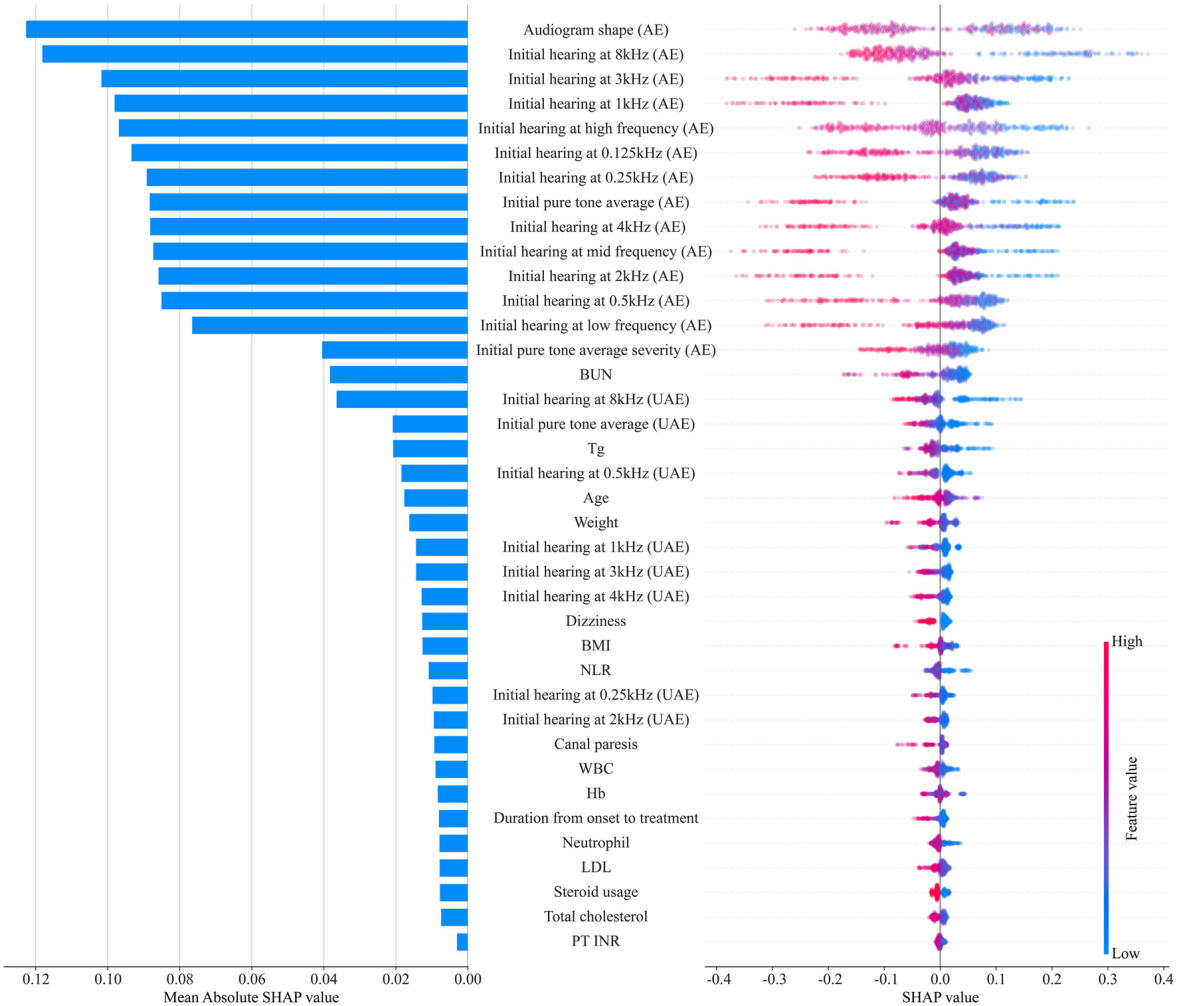
The feature importance bar plot and the SHAP summary plot. The left bar plot represents the importance of the variables with their overall contribution to the model prediction. The right dot plot represents the directionality with the contribution of the individual values for each variable. The red color indicates larger values, while the blue color indicates lower values for each variable. *SHAP* SHapley Additive exPlanation, *AE* affected ear, *BUN* blood urea nitrogen, *UAE* unaffected ear, *Tg* triglyceride, *BMI* body mass index, *NLR* neutrophil–lymphocyte ratio, *WBC* white blood cell, *Hb* hemoglobin, *LDL* low-density lipoprotein, *PT* prothrombin time, *INR* international normalized ratio.

This study included 453 patients with unilateral ISSNHL, including 250 who did not recover and 203 who did recover. The mean age was 50.3 years, and 220 patients (48.6%) were men. The features selected using recursive feature elimination and other clinical characteristics of patients according to their recovery status are listed in Table 1. A list of 38 selected features is presented according to feature importance (Fig. 2).

**Table 1:** Performance of the models in the test set.

Model	AUROC [95% CI]	AUPRC [95% CI]	Brier score	BACC	F1 score	*P* value^†^
**Baseline model**						
LogReg	0.813 [0.737–0.889]	0.786 [0.683–0.881]	0.176	0.692	0.667	
**Machine learning models**						
XGBoost	0.876 [0.812–0.939]	0.830 [0.707–0.933]	0.143	0.781	0.762	0.0242
SVM	0.892 [0.835–0.949]	0.862 [0.774–0.946]	0.135	0.768	0.740	0.0705
LightGBM	0.915 [0.864–0.967]	0.890 [0.814–0.962]	0.117	0.840	0.824	0.0015*
MLP	0.911 [0.859–0.964]	0.897 [0.818–0.958]	0.120	0.823	0.804	0.0010*

*AUPRC* area under the precision-recall curve, *AUROC* area under the receiver operating characteristic curve, *BACC* balanced accuracy, *LogReg* logistic regression, *MLP* multilayer perceptron, *SVM* support vector machine.

^†^AUROC comparison to logistic regression.

*P < 0.005.

### Model performance results

**Figure 3:**
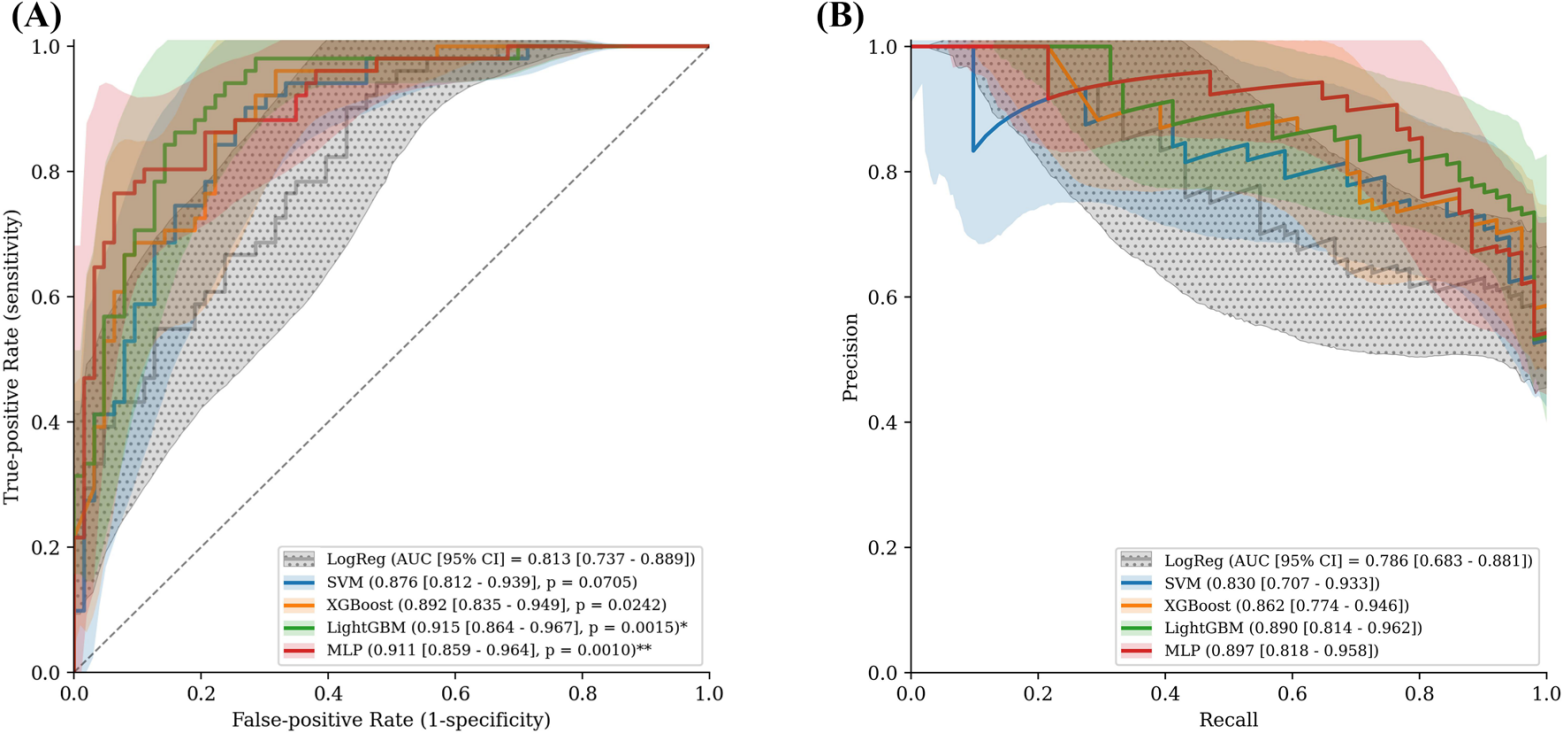
Performance results of the models. (**A**) Receiver operating characteristic (ROC) curve, (**B**) Precision-recall curve (PRC). Shades represent 95% confidence intervals (CI), and only the CIs of logistic regression (“LogReg”) are represented with polka dot patterns. Asterisk (*) indicates a significant difference compared to the logistic regression. *AUC* area under curve, *CI* confidence interval, *LogReg* logistic regression, *SVM* support vector machine, *XGBoost* extreme gradient boosting, *LightGBM* light gradient boosting machine, *MLP* multilayer perceptron.

The LightGBM achieved an AUROC CI of 0.915 (95% CI 0.864–0.967) and a balanced accuracy (BACC) of 0.84. The MLP achieved an AUROC of 0.911 (95% CI 0.859–0.964) and a BACC of 0.823. We confirmed that LightGBM and MLP were significantly superior to conventional LR as regards AUROC. SVM and XGBoost showed relatively higher AUROC values than the conventional LR, however these were not statistically significant. Likewise, the statistical superiority between the machine learning methods was not significant. Performance results in the test set are shown in Table 1 and Fig. 3. Cross-validation results are shown in Supplementary Table S3.

### Feature importance evaluation

The feature importance bar plot evaluated using the SHAP value is shown in Fig. 2. The impact of each feature on the predictive models was expressed as a bar plot of the mean absolute SHAP value. The plot reveals that the top 14 most important variables contributing to the model were associated with the initial hearing thresholds of the affected ear. The next most important features include laboratory data, such as blood urea nitrogen (BUN) and serum triglycerides (Tg), variables associated with the initial hearing thresholds of the unaffected ear, and demographic data such as age and weight. We also depicted the SHAP summary plot (Fig. 2), which shows how the high and low feature values were related to the SHAP values in the data set. Each dot represents the SHAP and feature values of each patient.

**Figure 4:**
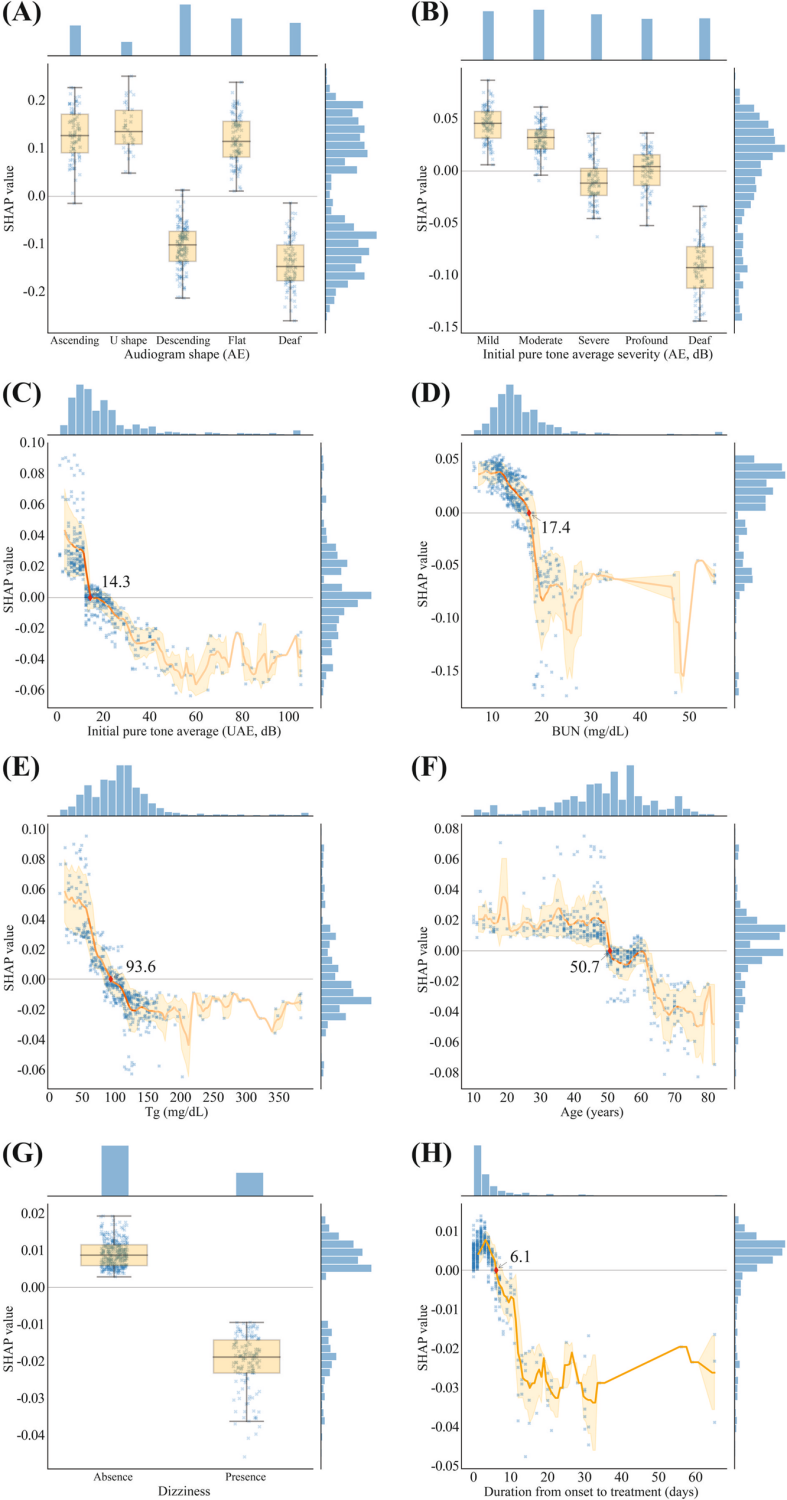
SHAP dependence plots for representative variables. (**A**) Audiogram shape (AE), (**B**) Initial pure tone average severity (AE, dB), (**C**) Initial pure tone average (UAE, dB), (**D**) BUN (mg/dL), (**E**) Tg (mg/dL), (**F**) Age (years), (**G**) Dizziness, (**H**) Duration from onset to treatment (days). Each variable was plotted on a scatter plot and a box plot with whiskers of 1.5 times the interquartile ranges (**A**, **B**, **G**) or a regression line with an orange line of mean and the shade of SD (**C**–**F**, **H**). The distributions of SHAP and variable values are represented with a histogram on the right and top of each plot. *SHAP* SHapley Additive exPlanation, *AE* affected ear, *BUN* blood urea nitrogen, *UAE* unaffected ear, *Tg* triglyceride.

The SHAP dependence plots (Fig. 4 and Supplementary Fig. S1) were also used to identify how a single feature affects model prediction. The y-axis values indicate the SHAP values of features and the x-axis values indicate the feature values. SHAP values for specific features above zero indicate a positive influence on model prediction (hearing recovery).

## Discussion

Artificial intelligence has been widely applied in various medical fields. It supports diagnosis with imaging processes (e.g., radiography in malignancy diagnosis) and predicts the prognosis of patients in intensive care^34–37^. It has been applied in various ways in the field of audiology. For example, it has been used to predict hearing loss in noise-exposed industrial workers. It has also been applied to auditory brainstem response or audiogram classification with good results^10–12,37–39^. However, these studies did not evaluate the contributions of the features. They had limitations not only in the performance of the models but also in how the models were interpreted. Recently, several techniques for the prognosis prediction of ISSNHL using artificial intelligence have been suggested^10–12^. We applied the SHAP method to evaluate the importance of the features. This allowed an evaluation of the contribution of each value of the variables. This could provide insights related to clinical decisions^36,40^. Although similar models have been used in several studies, the performance of the applied models vary widely. In this study, we developed and validated artificial intelligence methods including machine learning and deep learning models. These models were used to predict the prognosis of ISSNHL with a total of 38 features. Furthermore, the prognostic factors of ISSNHL were analyzed in a sophisticated manner. The results in our current study are superior to those of other studies, including our previous study. We believe this is due to the effective selection of features and a more advanced method design^15^. The predictive model using artificial intelligence will help clinicians to provide objective and quantifiable decisions. The SHAP method enables an evaluation of the individual value of the variables as well as contributes to the concept of personalized medicine.

The LightGBM and the MLP achieved a significantly higher AUROC score than the conventional statistical model. This is unlike the other machine learning models. LightGBM can natively manage categorical variables. This could be the reason for the higher AUROC than the baseline model. The results showed relatively high contributions for model predictions of some categorical variables, such as the audiogram shape and the initial pure tone average severity. The other models require one-hot encoding of the categorical data, which increases the number of features and makes the models vulnerable to overfitting problems. In addition, LightGBM implements a leaf-wise growth algorithm that splits a leaf node with maximum delta loss and minimizes the training loss. MLP also showed good performance in the AUROC in the cross-validation results (Supplementary Table S3). This study demonstrates that the neural network might act as a good feature extractor in our ISSNHL data. Therefore, we suggest that the advanced neural network architecture, which increases training efficiency and reduces overfitting problems, could lead to enhanced performance.

Many studies on the prognostic factors of ISSNHL have been and continue to be performed^1,3,7,41^. These prognostic factors include the involvement of medical factors, such as diabetes mellitus; laboratory factors, such as total cholesterol and LDL; inner ear factors, such as severity of initial hearing loss, audiogram shape, and presence of vertigo; treatment factors such as steroid usage and intratympanic steroid injection; and demographic factors such as age^2,3,5–7,38,42^. Predictors directly affecting model performance and the well-known prognostic factors should be carefully considered as the forecasters for prediction models. We evaluated the importance of all included variables at the same time using recursive feature elimination with a hierarchical double clustering scheme. This strategy minimized the underestimation of the importance of correlated variables that shared information with each other. It also allowed the selection of the most promising feature set in this study.

In this study, the initial audiogram shape had the top feature importance. The influence of the audiogram shape on the prognosis could be shown through the SHAP dependence plot (Fig. 4). The ascending, U-shaped and flat types have positive mean SHAP values. This means that they have a positive effect on prognosis. However, descending and deaf types showing negative mean SHAP values have a negative effect on the prognosis. Our findings strongly suggest that high-frequency hearing loss could affect the prognosis of ISSNHL. Damage to the basal end of the cochlea is an important recovery factor^43^. These results are consistent with those of previous studies^43,44^. The suggested reasons for the poorer recovery of the basal end of cochlea, which governs high-frequency hearing, includes its functional metabolic needs and the blood supply between the apex and the base.

The next important feature was the initial hearing result. This variable has a higher SHAP value for mild to moderate hearing loss and a lower mean SHAP value for severe to profound hearing loss or deafness. This implies that mild to moderate hearing loss has a better prognosis. Previous studies have shown similar results. We believe that the more severe the inflammation or damage to the inner ear, the slower and less extensive hearing recovery^2,43,44^.

Interestingly, initial hearing levels of the unaffected ear were also important for the prognosis. The poorer they were, the less the hearing recovery. This phenomenon might indicate that the entire hearing system of ISSNHL is poor, or that the potential for recovery remains low^2,44^.

To the best of our knowledge, no study has identified serum BUN as a prognostic factor. However, our results showed a decrease of SHAP values with a cut-off value of 17.4 according to an increase in BUN. This can be interpreted based on the theory that the pathogenesis of ISSNHL is the microvascular occlusion of the inner ear. In general, BUN is related to volume status, and high BUN levels indicate dehydration and low blood flow. Therefore, it can be hypothesized that the prognosis is poor in patients with high BUN levels because of low blood flow to the inner ear. Hence, hydration can be regarded as helpful in the prognosis of ISSNHL.

Tg could be a poor prognostic factor as seen when the value was high. This denoted a negative mean SHAP value with a cut-off value of 93.6. The association between comorbid dyslipidemia and hearing improvement in patients with ISSNHL is controversial. Certain reports claim it has a negative effect on prognosis, whereas others do not^45–49^. The hypothesis for a poor prognosis is that hyperlipidemia may augment microvascular insufficiency in ISSNHL, resulting in a poor prognosis^45^. Regarding age, older patients tend to have negative mean SHAP values, as the cut-off value was 50.7. This means that older patients tend to have a poorer prognosis. The prognosis was poor for those over 60 years old because immune defense mechanisms deteriorate as patients age^2^. Dizziness had a negative mean SHAP value, which indicates a negative effect on prognosis. When ISSNHL is accompanied by dizziness, it has a detrimental effect on ISSNHL. Whether it includes widespread inflammation or vascular ischemia, these conditions can be considered to be extensive in the inner ear. Our findings are consistent with those of previous studies^5,9,13,50,51^.

The duration from onset to treatment is known to be a prognostic factor. Early treatment had a positive mean SHAP value. Its cut-off value was 6.1 days. Early treatment can be interpreted as having a good effect on prognosis if treatment is initiated earlier than 6.1 days from onset. The timing of treatment is controversial. Several previous studies reported that patients who began treatment within 1 week after hearing loss had a high hearing recovery rate. However, there are reports that the beginning of treatment and prognosis are not related^2,16,43^. However, we believe that early treatment has a good prognosis. Most of the other features also showed meaningful patterns in the SHAP dependence plots (Supplementary Fig. S1).

This study has some limitations. First, the sample size was relatively small, and machine learning performance largely depends on the sample size. LightGBM and MLP which presented higher performance than baseline model in this study, are generally prone to overfitting. Future studies should verify the algorithms with larger sample sizes. Second, to include various features, we used the missing value imputation method to replace missing feature values. Replaced values cannot fully reflect actual values; moreover, they can affect the performance of the models.

In conclusion, our machine and deep learning models showed superior performance in predicting the prognosis of ISSNHL. In particular, the LightGBM presented the highest predictive power and the lowest prediction error. Through further studies with large sample sizes and methodological improvements, we believe that artificial intelligence, including our models, can be applied to patients to predict ISSNHL prognosis and enhance clinical effectiveness.

## Supplementary Information

Supplementary Figure S1.

Supplementary Table S1.

Supplementary Table S2.

Supplementary Table S3.
