# Supplementary material for: Prediction of hearing recovery in unilateral sudden sensorineural hearing loss using artificial intelligence
Source: Sci Rep. 2022 Mar 10;12:3977. doi: 10.1038/s41598-022-07881-2 (PMC8913667; doi:10.1038/s41598-022-07881-2)
Supplement: Supplementary file 1 — Supplementary Figure S1. [file 41598_2022_7881_MOESM1_ESM.pdf]

Supplementary Figure S1. Partial SHAP Dependence Plots for the Selected Variables

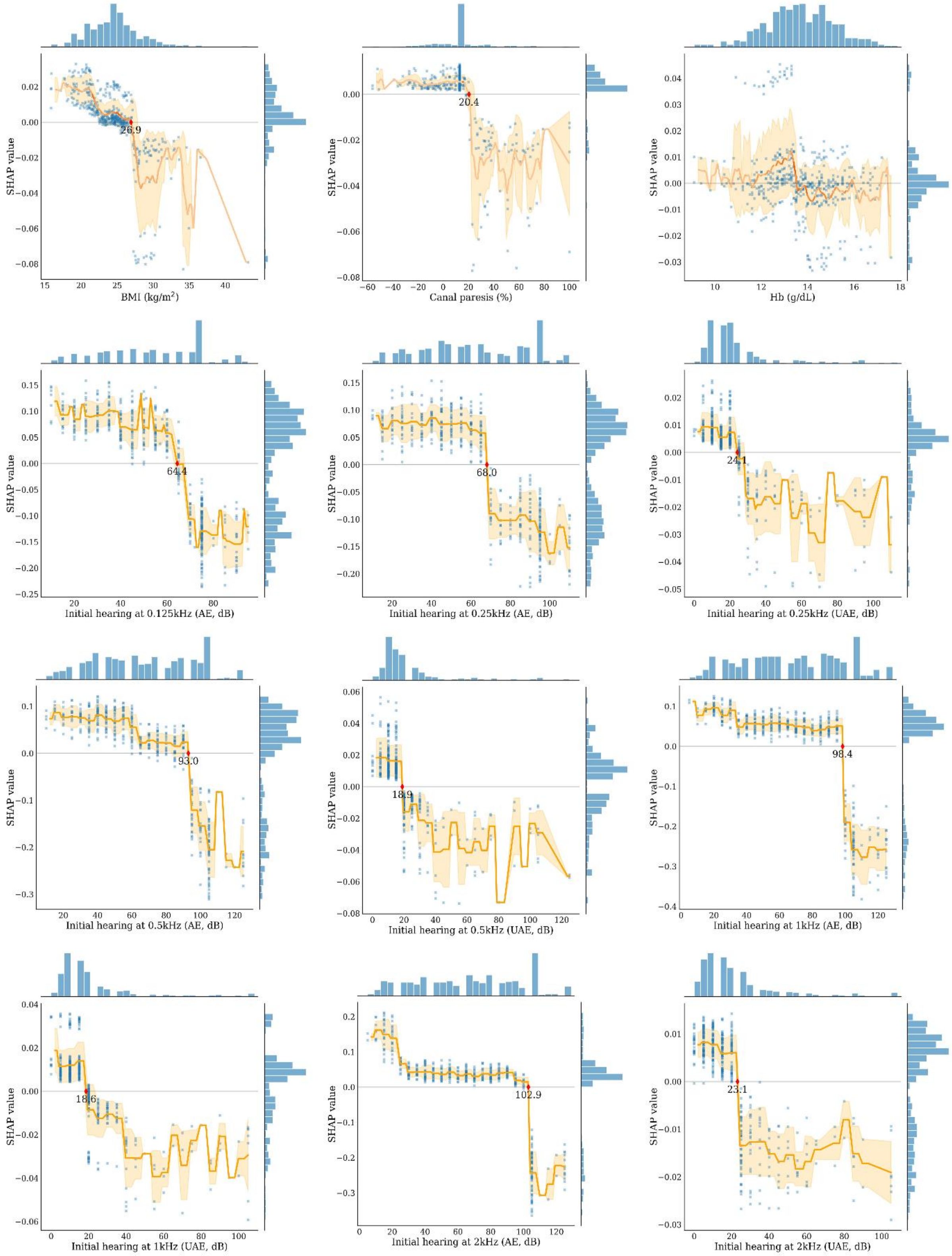

Supplementary Figure S1. *(Continued.)*

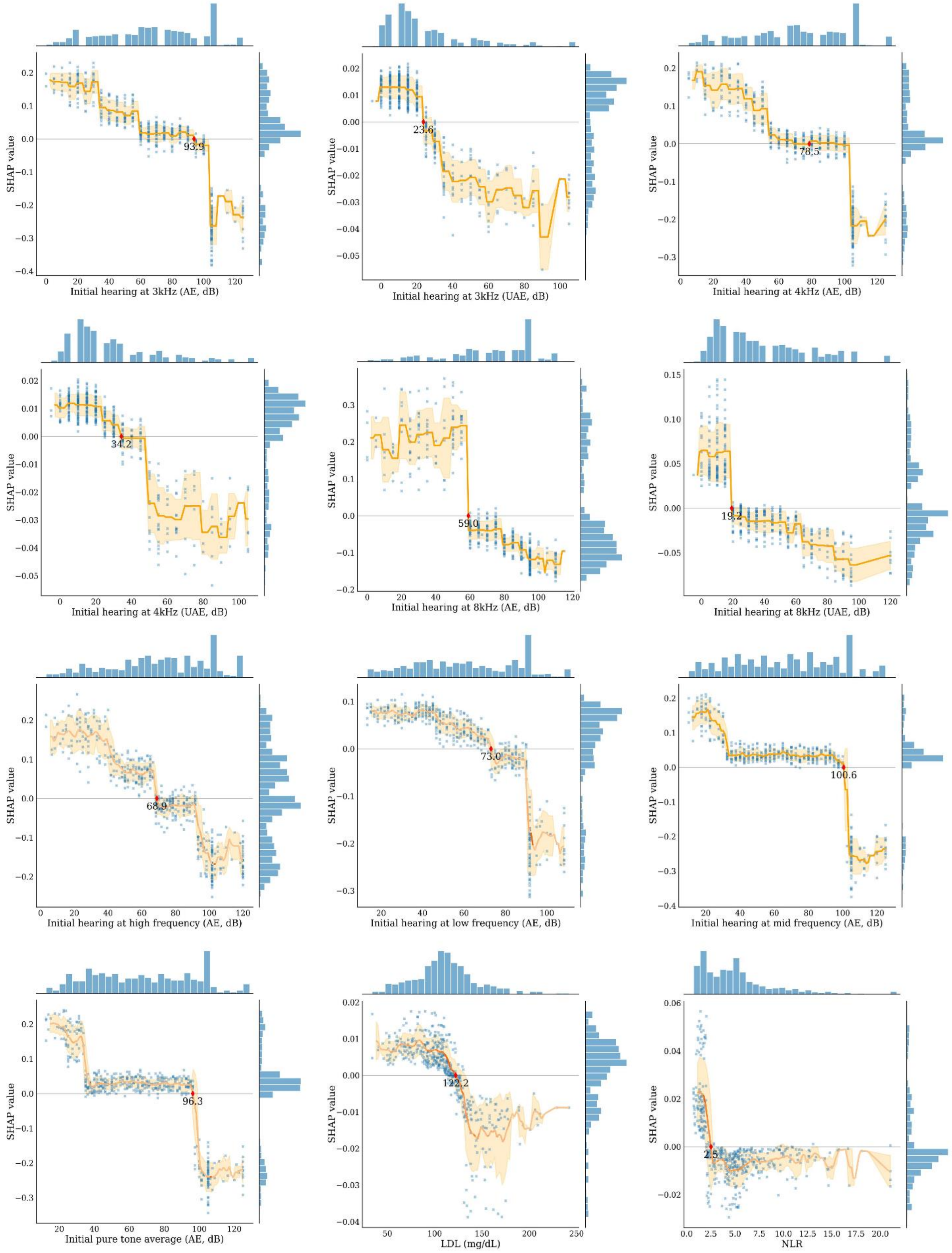

Supplementary Figure S1. *(Continued.)*

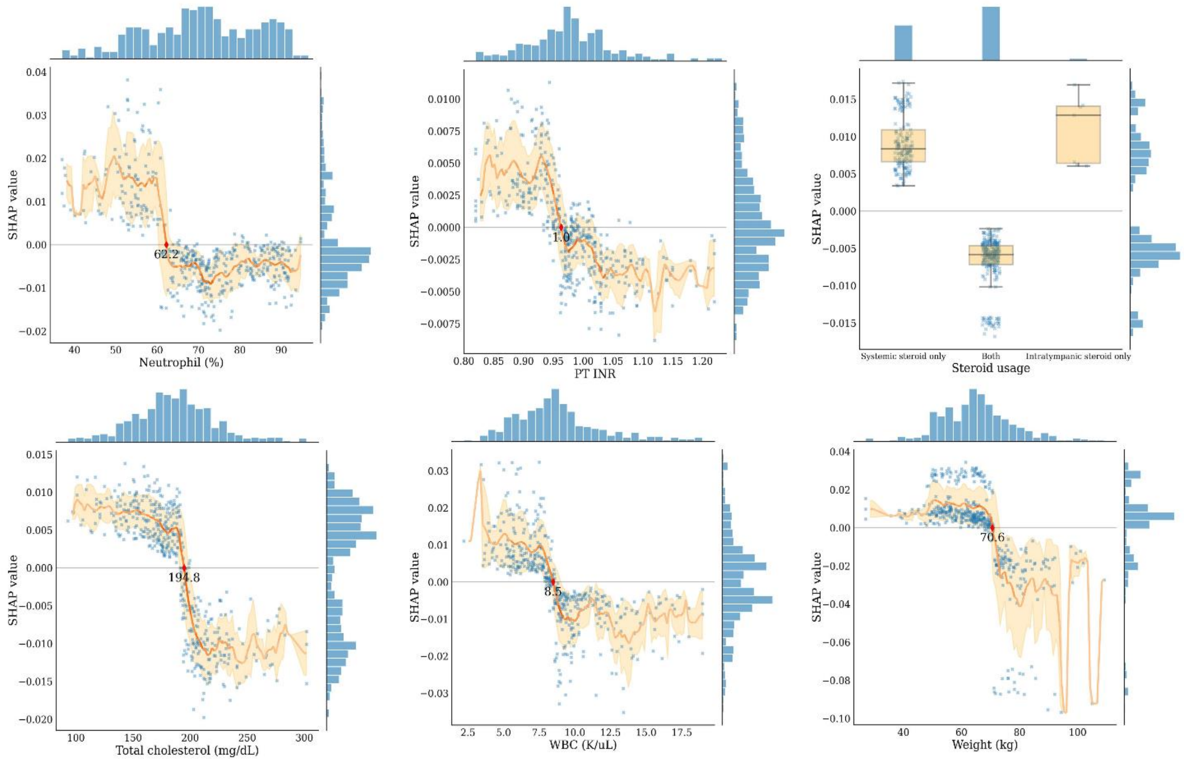

BMI, body mass index; HTN, hypertension; DM, diabetes mellitus; CKD, chronic kidney disease; MI, myocardial infarction; Hb, hemoglobin; BUN, blood urea nitrogen; Tg, triglyceride; LDL, low-density lipoprotein; WBC, white blood cell; NLR, neutrophil-lymphocyte ratio; PT, prothrombin time; INR, international normalized ratio.
