# Supplementary material for: Prediction of hearing recovery in unilateral sudden sensorineural hearing loss using artificial intelligence
Source: Sci Rep. 2022 Mar 10;12:3977. doi: 10.1038/s41598-022-07881-2 (PMC8913667; doi:10.1038/s41598-022-07881-2)
Supplement: Supplementary file 2 — Supplementary Table S1. [file 41598_2022_7881_MOESM2_ESM.docx]

**Supplementary Table S1.** Clinical Characteristics and Features for Patients with and without Recovery

| **Variables** | | **No recovery (n=250)** | | **Recovery (n=203)** | | ***p*** | |
| --- | --- | --- | --- | --- | --- | --- | --- |
| Sex, male: female | | 129:121 (51.6%:48.4%) | | 91:112 (44.8%:55.2%) | | 0.180 | |
| Age at the time of diagnosis (years) | | 53.0 (46.0, 61.0) | | 47.0 (37.0, 55.5) | | **< 0.001** | |
| Affected side, right: left | | 112:138 (44.8%:55.2%) | | 99:104 (48.8%:51.2%) | | 0.455 | |
| BMI, kg/m^2^ | | 24.9 (3.9) | | 24.0 (3.6) | | **0.013** | |
| Weight, kg | | 66.3 (13.1) | | 63.8 (12.1) | | **0.044** | |
| Duration from onset to treatment, days | | 2.0 (1.0, 6.0) | | 3.0 (1.0, 5.0) | | 0.385 | |
| Comorbidity | |  | |  | |  | |
| HTN | | 89 (35.6%) | | 35 (17.2%) | | **< 0.001** | |
| DM | | 76 (30.4%) | | 36 (17.7%) | | **0.003** | |
| Dyslipidemia | | 17 (6.8%) | | 11 (5.4%) | | 0.701 | |
| Stroke | | 3 (1.2%) | | 2 (1.0%) | | 0.806 | |
| CKD | | 9 (3.6%) | | 2 (1.0%) | | 0.140 | |
| MI/Angina | | 15 (6.0%) | | 4 (2.0%) | | 0.061 | |
| Initial hearing threshold of affected ear, dB | |  | |  | |  | |
| 0.125 kHz | | 58.7 (21.2) | | 47.4 (20.3) | | **< 0.001** | |
| 0.25 kHz | | 70.0 (45.0, 95.0) | | 50.0 (37.5, 67.5) | | **< 0.001** | |
| 0.5 kHz | | 75.0 (50.0, 100.0) | | 55.0 (40.0, 75.0) | | **< 0.001** | |
| 1 kHz | | 80.0 (50.0, 105.0) | | 55.0 (32.5, 80.0) | | **< 0.001** | |
| 2 kHz | | 80.0 (50.0, 105.0) | | 55.0 (25.0, 77.5) | | **< 0.001** | |
| 3 kHz | | 77.6 (28.6) | | 53.5 (28.3) | | **< 0.001** | |
| 4 kHz | | 85.0 (60.0, 105.0) | | 55.0 (30.0, 80.0) | | **< 0.001** | |
| 8 kHz | | 90.0 (70.0, 95.0) | | 60.0 (35.0, 85.0) | | **< 0.001** | |
| Low frequency | | 70.8 (46.7, 91.7) | | 50.0 (38.3, 68.3) | | **< 0.001** | |
| Mid frequency | | 80.0 (50.0, 105.0) | | 55.0 (32.5, 77.5) | | **< 0.001** | |
| High frequency | | 85.0 (62.1, 101.7) | | 60.0 (32.5, 78.3) | | **< 0.001** | |
| Average | | 77.5 (49.1, 105.0) | | 55.0 (32.5, 77.5) | | **< 0.001** | |
| Severity | |  | |  | |  | |
| Mild, 20–39 dB | | 33 (13.2%) | | 64 (31.5%) | | **< 0.001** | |
| Moderate, 40–59 dB | | 50 (20.0%) | | 50 (24.6%) | | 0.286 | |
| Severe, 60–79 dB | | 48 (19.2%) | | 43 (21.2%) | | 0.685 | |
| Profound, 80–100 dB | | 41 (16.4%) | | 41 (20.2%) | | 0.357 | |
| Deaf, 100 dB | | 78 (31.2%) | | 5 (2.5%) | | **< 0.001** | |
| **Supplementary Table S1.** *(Continued.)* | | | | | | |  |
| **Variables** | **No recovery (n=250)** | | **Recovery (n=203)** | | ***p*** | |  |
| Audiogram shape of affected ear |  | |  | |  | |  |
| Ascending | 30 (12.0%) | | 53 (26.1%) | | **< 0.001** | |  |
| U shape | 11 (4.4%) | | 27 (13.3%) | | **0.001** | |  |
| Descending | 84 (33.6%) | | 56 (27.6%) | | 0.202 | |  |
| Flat | 43 (17.2%) | | 59 (29.1%) | | **0.004** | |  |
| Deaf | 82 (32.8%) | | 8 (3.9%) | | **< 0.001** | |  |
| Initial hearing threshold of unaffected ear, dB |  | |  | |  | |  |
| 0.125 kHz | 24.7 (17.4) | | 20.1 (12.9) | | **0.002** | |  |
| 0.25 kHz | 20.0 (15.0, 30.0) | | 15.0 (10.0, 20.0) | | **< 0.001** | |  |
| 0.5 kHz | 15.0 (10.0, 25.0) | | 15.0 (10.0, 20.0) | | **< 0.001** | |  |
| 1 kHz | 15.0 (10.0, 30.0) | | 15.0 (10.0, 20.0) | | **< 0.001** | |  |
| 2 kHz | 20.0 (10.0, 30.0) | | 10.0 (5.0, 20.0) | | **< 0.001** | |  |
| 3 kHz | 22.5 (10.0, 43.8) | | 15.0 (5.0, 20.0) | | **< 0.001** | |  |
| 4 kHz | 30.0 (15.0, 55.0) | | 15.0 (10.0, 30.0) | | **< 0.001** | |  |
| 8 kHz | 40.0 (20.0, 70.0) | | 20.0 (10.0, 40.0) | | **< 0.001** | |  |
| Average | 18.8 (12.5, 32.5) | | 13.8 (8.8, 20.0) | | **< 0.001** | |  |
| Severity |  | |  | |  | |  |
| Mild, 20–39 dB | 205 (82.0%) | | 192 (94.6%) | | **< 0.001** | |  |
| Moderate, 40–59 dB | 23 (9.2%) | | 6 (3.0%) | | **0.012** | |  |
| Severe, 60–79 dB | 7 (2.8%) | | 2 (1.0%) | | 0.299 | |  |
| Profound, 80–100 dB | 8 (3.2%) | | 3 (1.5%) | | 0.380 | |  |
| Deaf, 100 dB | 7 (2.8%) | | 0 (0.0%) | | **0.043** | |  |
| Smoking status |  | |  | |  | |  |
| Never | 206 (82.4%) | | 164 (80.8%) | | 0.750 | |  |
| Current | 37 (14.8%) | | 29 (14.3%) | | 0.984 | |  |
| ex-smoker | 7 (2.8%) | | 8 (3.9%) | | 0.681 | |  |
| Dizziness | 105 (42.0%) | | 37 (18.2%) | | **< 0.001** | |  |
| Steroid usage |  | |  | |  | |  |
| Systemic steroid only | 82 (32.8%) | | 94 (46.3%) | | **0.005** | |  |
| Intratympanic steroid only | 2 (0.8%) | | 5 (2.5%) | | 0.296 | |  |
| Both | 166 (66.4%) | | 104 (51.2%) | | **0.001** | |  |
| Hb, g/dL | 13.6 (1.6) | | 13.7 (1.5) | | 0.818 | |  |
| BUN, mg/dL | 17.4 (8.7) | | 14.5 (6.7) | | **< 0.001** | |  |
| Total cholesterol, mg/dL | 188.1 (42.3) | | 182.4 (41.8) | | 0.249 | |  |
| Tg, mg/dL | 126.1 (87.4) | | 93.0 (67.2) | | **< 0.001** | |  |

**Supplementary Table S1.** *(Continued.)*

| **Variables** | **No recovery (n=250)** | **Recovery (n=203)** | ***p*** |
| --- | --- | --- | --- |
| LDL, mg/dL | 113.3 (41.8) | 105.0 (36.9) | 0.092 |
| WBC, ×10^3^/μL | 8.7 (3.3) | 9.0 (3.7) | 0.434 |
| Neutrophil, % | 71.0 (15.2) | 70.6 (15.4) | 0.840 |
| NLR | 5.2 (4.5) | 5.0 (4.1) | 0.769 |
| PT, INR | 1.0 (0.1) | 1.0 (0.1) | 0.494 |
| Canal paresis, % | 17.7 (33.0) | 6.6 (27.6) | **0.013** |
| Neutrophil, % | 71.0 (15.2) | 70.6 (15.4) | 0.840 |
| NLR | 5.2 (4.5) | 5.0 (4.1) | 0.769 |
| PT, INR | 1.0 (0.1) | 1.0 (0.1) | 0.494 |
| Canal paresis, % | 17.7 (33.0) | 6.6 (27.6) | **0.013** |
| a) Categorical variables are presented as number (percentage). Continuous variables that follow normal distribution are presented as mean (standard deviation). Continuous variables that do not follow normal distribution are presented as median values (25% quartiles, 75% quartiles).  b) Owing to missing values, the total number of some variables are different. The number of missing values for each variable are listed in Supplementary Table S2.  BMI, body mass index; HTN, hypertension; DM, diabetes mellitus; CKD, chronic kidney disease; MI, myocardial infarction; Hb, hemoglobin; BUN, blood urea nitrogen; Tg, triglyceride; LDL, low-density lipoprotein; WBC, white blood cell; NLR, neutrophil-lymphocyte ratio; PT, prothrombin time; INR, international normalized ratio. | | | |
