# Supplementary material for: Prediction of hearing recovery in unilateral sudden sensorineural hearing loss using artificial intelligence
Source: Sci Rep. 2022 Mar 10;12:3977. doi: 10.1038/s41598-022-07881-2 (PMC8913667; doi:10.1038/s41598-022-07881-2)
Supplement: Supplementary file 3 — Supplementary Table S2. [file 41598_2022_7881_MOESM3_ESM.pdf]

Supplementary Table S2. Variables Type and Missing Rates of the Data

Variables	Types	Missing rates
Sex	categorical variable	0.0%
Age	continuous variable	0.0%
BMI	continuous variable	6.8%
Weight	continuous variable	6.8%
Duration from onset to treatment	continuous variable	0.2%
Smoking status	categorical variable	0.4%
Smoking (years)	continuous variable	0.4%
Steroid usage	categorical variable	0.0%
Comorbidity		
HTN	categorical variable	0.4%
DM	categorical variable	0.4%
Dyslipidemia	categorical variable	0.4%
Stroke	categorical variable	0.4%
CKD	categorical variable	0.4%
MI/Angina	categorical variable	0.4%
Tinnitus	categorical variable	0.4%
Dizziness	categorical variable	0.4%
ESR	continuous variable	45.3%
PT_sec	continuous variable	16.3%
PT_p	continuous variable	16.3%
aPTT	continuous variable	16.3%
Cr	continuous variable	8.4%
Platelet	continuous variable	24.9%
Hb	continuous variable	9.1%
BUN	continuous variable	8.6%
Total cholesterol	continuous variable	34.4%
Tg	continuous variable	40.4%
LDL	continuous variable	41.7%
WBC	continuous variable	24.9%
Neutrophil	continuous variable	24.9%
NLR	continuous variable	24.9%
PT(INR)	continuous variable	60.0%

Supplementary Table S2. *(Continued.)*

Variables	Types	Missing rates
Canal paresis	continuous variable	53.9%
Initial hearing threshold of affected ear		
0.125 kHz	continuous variable	3.3%
0.25 kHz	continuous variable	0.2%
0.5 kHz	continuous variable	0.0%
1 kHz	continuous variable	0.0%
2 kHz	continuous variable	0.0%
3 kHz	continuous variable	0.4%
4 kHz	continuous variable	0.0%
8 kHz	continuous variable	0.7%
Low frequency	continuous variable	0.0%
Mid frequency	continuous variable	0.0%
High frequency	continuous variable	0.0%
Average	continuous variable	0.0%
Severity	categorical variable	0.0%
Audiogram shape of affected ear	categorical variable	0.0%
Initial hearing threshold of unaffected ear		
0.125 kHz	continuous variable	2.6%
0.25 kHz	continuous variable	0.2%
0.5 kHz	continuous variable	0.0%
1 kHz	continuous variable	0.0%
2 kHz	continuous variable	0.0%
3 kHz	continuous variable	0.0%
4 kHz	continuous variable	0.2%
8 kHz	continuous variable	0.2%
Average	continuous variable	0.0%
Severity	categorical variable	0.0%
Pre-SDS_affected	continuous variable	50.6%
Pre-SDS_unaffected	continuous variable	50.3%
c_VEMP_abn	categorical variable	72.4%
o_VEMP_abn	categorical variable	73.1%
PRE_F	continuous variable	60.5%
PRE_E	continuous variable	60.5%
PRE_C	continuous variable	60.5%
PRE_Total	continuous variable	60.5%

BMI, body mass index; HTN, hypertension; DM, diabetes mellitus; CKD, chronic kidney disease; MI, myocardial infarction; Hb, hemoglobin; BUN, blood urea nitrogen; Tg, triglyceride; LDL, low-density lipoprotein; WBC, white blood cell; NLR, neutrophil-lymphocyte ratio; PT, prothrombin time; INR, international normalized ratio.
