# Supplementary material for: Prediction of hearing recovery in unilateral sudden sensorineural hearing loss using artificial intelligence
Source: Sci Rep. 2022 Mar 10;12:3977. doi: 10.1038/s41598-022-07881-2 (PMC8913667; doi:10.1038/s41598-022-07881-2)
Supplement: Supplementary file 4 — Supplementary Table S3. [file 41598_2022_7881_MOESM4_ESM.pdf]

Supplementary Table S3. Cross-Validation Results in the Training Set

Model	AUROC	AUPRC	Brier score	BACC	F1 score
Baseline model					
LogReg	0.882 (0.207)	0.934 (0.114)	0.06 (0.103)	0.872 (0.218)	0.743 (0.437)
Machine learning models					
XGBoost	0.891 (0.048)	0.89 (0.051)	0.117 (0.031)	0.815 (0.062)	0.787 (0.085)
SVM	0.903 (0.056)	0.898 (0.061)	0.105 (0.040)	0.833 (0.074)	0.807 (0.100)
LightGBM	0.928 (0.036)	0.923 (0.039)	0.094 (0.030)	0.854 (0.053)	0.834 (0.065)
MLP	0.942 (0.027)	0.941 (0.030)	0.084 (0.024)	0.871 (0.040)	0.854 (0.048)

The values are aggregated cross-validation scores in all folds and expressed as mean (SD).
AUPRC, area under the precision-recall curve; AUROC, area under the receiver operating characteristic curve; BACC, balanced accuracy; LogReg, logistic regression; MLP multilayer perceptron; SVM, support vector machine.
